# High-throughput screening identifies a trafficking corrector for long QT syndrome–associated KCNQ1 variants

**DOI:** 10.1172/jci.insight.201297

**Published:** 2026-01-08

**Authors:** Katherine R. Clowes Moster, Carlos G. Vanoye, Ana C. Chang-Gonzalez, Ian M. Romaine, Katherine M. Stefanski, Mason C. Wilkinson, Joshua A. Bauer, Thomas P. Hasaka, Emily L. Days, Reshma R. Desai, Kathryn R. Butcher, Gary A. Sulikowski, Alex G. Waterson, Jens Meiler, Kaitlyn V. Ledwitch, Alfred L. George, Charles R. Sanders

**Affiliations:** 1 Department of Biochemistry and; 2 Center for Structural Biology, Vanderbilt University, Nashville, Tennessee, USA.; 3 Department of Pharmacology, Northwestern University Feinberg School of Medicine, Chicago, Illinois, USA.; 4 Department of Chemistry and; 5 Vanderbilt Institute of Chemical Biology, Vanderbilt University, Nashville, Tennessee, USA.; 6 Department of Pharmacology, Vanderbilt University School of Medicine, Nashville, Tennessee, USA.; 7 Center for Applied Artificial Intelligence in Protein Dynamics, Vanderbilt University, Nashville, Tennessee, USA.; 8 Institute for Drug Discovery, Faculty of Medicine, Faculty of Mathematics and Informatics, Faculty of Chemistry and Mineralogy, University Leipzig, Leipzig, Germany.; 9 Center for Scalable Data Analytics and Artificial Intelligence (ScaDS.AI) and School of Embedded Composite Artificial Intelligence (SECAI), Dresden/Leipzig, Germany.

**Keywords:** Cardiology, Genetics, Arrhythmias, Ion channels, Pharmacology

## Abstract

Congenital long QT syndrome (LQTS) promotes risk for life-threatening cardiac arrhythmia and sudden death in children and young adults. Pathogenic variants in the voltage-gated potassium channel KCNQ1 are the most frequently discovered genetic cause. Most LQTS-associated KCNQ1 variants cause loss of function secondary to impaired trafficking of the channel to the plasma membrane. There are currently no therapeutic approaches that address this underlying molecular defect. Using a high-throughput screening paradigm, we identified VU0494372, a small molecule that increases total and cell surface levels and trafficking efficiency of WT KCNQ1 as well as three LQTS-associated variants. Additionally, 16-hour treatment of cells with VU0494372 increased I__Ks__ (KCNQ1-KCNE1 current) for WT KCNQ1 and the LQTS-associated variant V207M in cells coexpressing KCNE1. VU0494372 had no impact on KCNQ1 transcription, degradation, or thermal stability, and increased the rate of KCNQ1 reaching the cell surface. We identified a potential direct interaction site with KCNQ1 at or near the binding site of the KCNQ1 potentiator ML277. Together, these findings demonstrate that small molecules can increase the expression levels and cell surface trafficking efficiency of KCNQ1 and introduce a potential new pharmacological approach for treating LQTS.

## Abstract 2

We discovered and investigated the first trafficking corrector for KCNQ1 mutations that cause congenital long QT syndrome (LQTS), a life-threatening cardiac arrhythmia.

## Graphical abstract

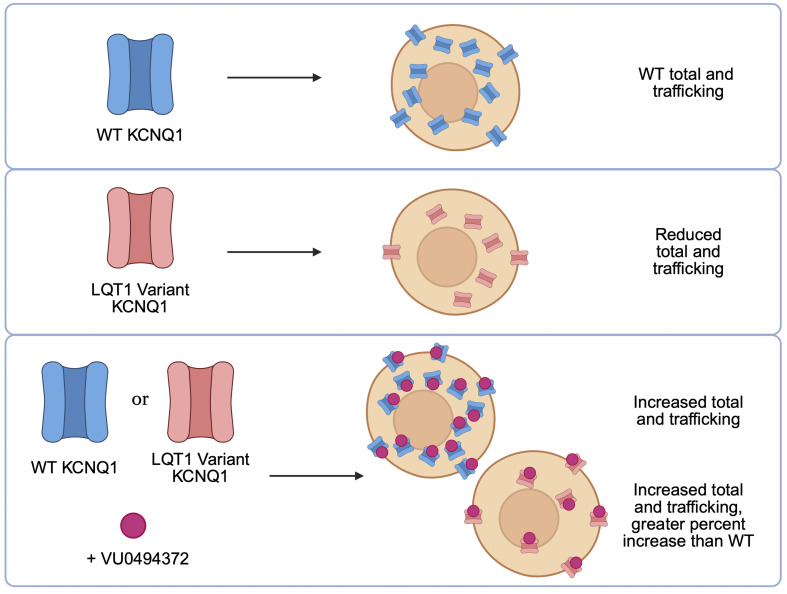

## Introduction

Long QT syndrome (LQTS) is a life-threatening disorder of heart rhythm caused by impaired repolarization of cardiac action potentials, reflected by an increase in duration of the QT interval in the electrocardiogram (1). LQTS is associated with syncope, cardiac arrhythmia, and cardiac arrest, which can be fatal (2). It is estimated that 1:2,000 to 1:2,500 individuals are affected by congenital LQTS, making it one of the most common monogenic disorders (3, 4).

Congenital LQTS is caused by pathogenic variants in many genes mostly encoding ion channels or ion channel modulators (5). Type 1 long QT syndrome (LQT1) accounts for up to 50% of cases of congenital LQTS and is caused by loss of function of the voltage-gated potassium channel KCNQ1 (also known as K__v__7.1 or K__v__LQT1) (5, 6). KCNQ1, together with its accessory protein KCNE1, mediates the slow delayed rectifier current (I__Ks__) that drives repolarization during the cardiac action potential (7). Hundreds of pathogenic KCNQ1 variants have been identified, and these variants are distributed throughout the protein sequence (8, 9). Clinical interventions for LQTS include avoidance of triggers such as strenuous exercise, administration of beta blockers, and surgical implantation of a cardioverter-defibrillator (10, 11). However, no treatments currently exist that target the underlying molecular defects in KCNQ1.

Previous studies have indicated that roughly half of known disease-associated variants disrupt KCNQ1 function through protein destabilization, which leads to failure to traffic to the plasma membrane (mistrafficking), in some cases followed by protein degradation (12, 13). In this regard, LQT1 resembles other disorders for which mistrafficking of a membrane protein drives disease mechanisms, including retinitis pigmentosa (rhodopsin photoreceptor) (14–18), diabetes insipidus (vasopressin 2 receptor) (19, 20), type 1E Charcot-Marie-Tooth disease (peripheral myelin protein 22 [PMP22]) (21–23), and cystic fibrosis (cystic fibrosis transmembrane regulator [CFTR]) (24–27). For some of these proteins, small molecules have been identified that bind to disease-associated variants to stabilize folding and increase cell surface levels (28–34). For example, in cystic fibrosis (CF), extensive research led to the development of a 3-drug therapy effective against the ΔF508 CFTR variant responsible for the majority of all CF cases (35–37). Two of these 3 drugs act by restoring normal folding and trafficking of the mutant protein (trafficking correctors), while the third enhances channel activity (potentiator) (32, 36–38). This treatment has now also been found to be effective for additional CFTR variants (39, 40). As many KCNQ1 variants appear to exhibit similar defects in folding and trafficking, we hypothesized that KCNQ1 dysfunction could be remedied in a similar manner to CFTR. While activators of KCNQ1 have been discovered (41–44), no compounds have yet been identified that modify KCNQ1 trafficking. Identification of a small molecule in this class would provide support for this hypothesis and provide proof-of-concept support for treating LQT1 by targeting KCNQ1 mistrafficking.

Here, we investigated whether cell surface levels of KCNQ1 and KCNQ1 trafficking efficiency can be pharmacologically enhanced using a high-throughput screen of chemically diverse small molecules. We identified the compound VU0494372, which increases KCNQ1 cell surface levels, total levels, and surface trafficking efficiency in a dose-dependent manner. This compound represents a first-in-class small-molecule modulator of KCNQ1 trafficking and provides a foundation for future LQT1 drug discovery efforts.

## Results

### *Discovery of KCNQ1 trafficking modulators with high-throughput screening*.

**Figure 1:**
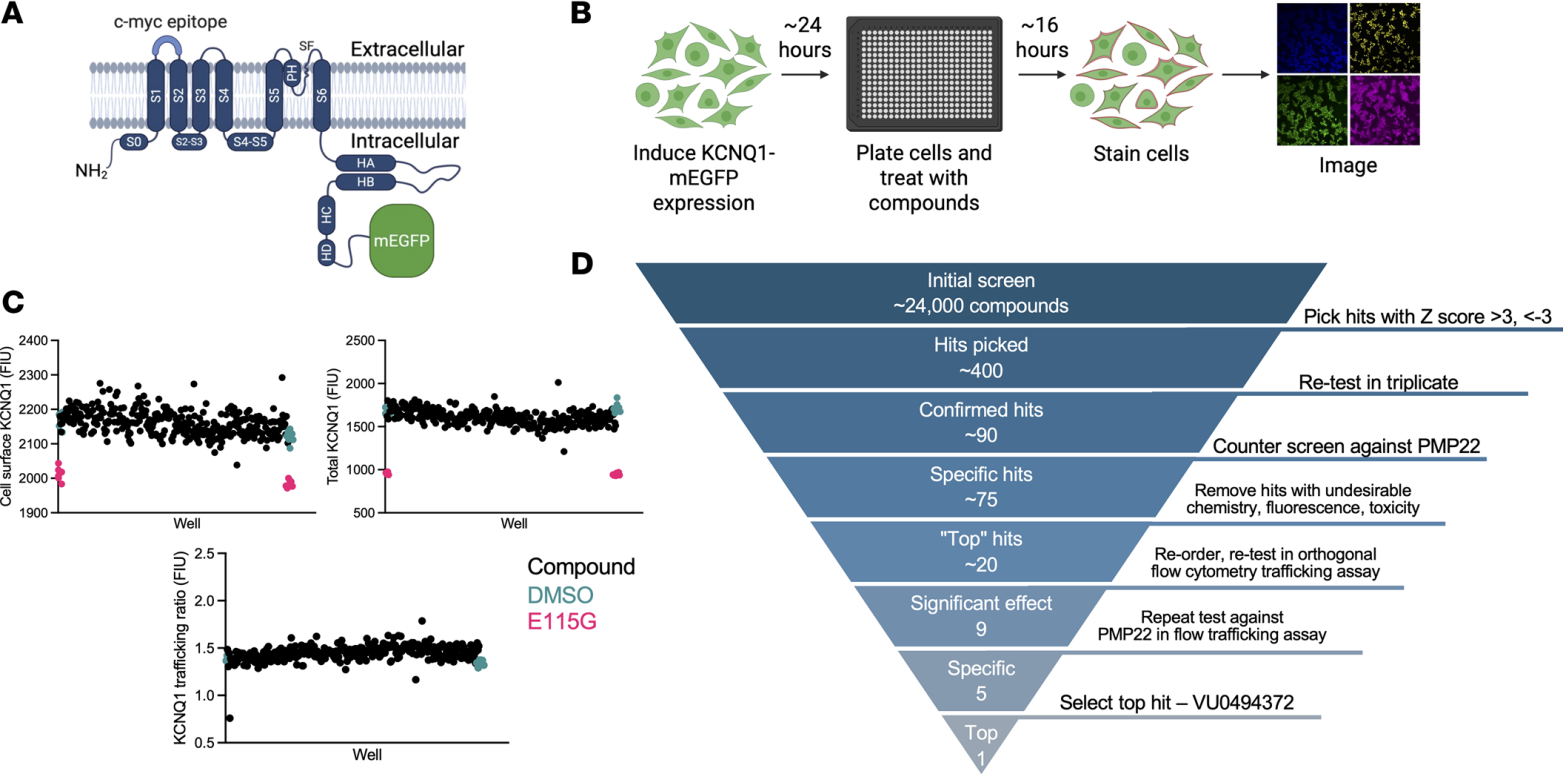
A high-throughput screen to identify compounds that alter KCNQ1 expression and/or trafficking. (**A**) KCNQ1 construct (mycKCNQ1-mEGFP) used for high-throughput screening. (Created in BioRender: Clowes K, 2026, https://BioRender.com/jqaizjl.) (**B**) Workflow for high-throughput screening. (Created in BioRender: Clowes K, 2026, https://BioRender.com/ctyod9v.) (**C**) Representative data showing quantifications of cell surface KCNQ1 (left), total cellular KCNQ1 (right), and KCNQ1 trafficking ratio (bottom) for a screened 384-well plate. Each point represents the average value for all quantified cells in one well. Black, compound-treated well; teal, DMSO-treated negative control well; magenta, E115G mycKCNQ1-mEGFP positive control well. E115G is not shown in the trafficking ratio plot because of skewing of values from near background-level surface and total levels. FIU, fluorescence intensity units. (**D**) Screening funnel summarizing the steps of the process for narrowing from about 24,000 screened compounds to the top hit VU0494372.

To conduct high-throughput screening for compounds that alter the expression and/or trafficking of KCNQ1 in mammalian cells, we developed a high-content image-based trafficking assay. We first generated a cell line that stably expresses KCNQ1 by utilizing “LLP-int” HEK293T cells, which contain a genomic “landing pad” DNA integration site under control of a tetracycline-inducible promoter where DNA of interest can be integrated (45) (Supplemental Figure 1A; supplemental material available online with this article; https://doi.org/10.1172/jci.insight.201297DS1). We inserted a cassette containing wild-type (WT) human mycKCNQ1-mEGFP cDNA, a KCNQ1 construct with a myc epitope inserted into the extracellular S1–S2 linker (46) and a monomeric enhanced green fluorescent protein (mEGFP) fused to the C-terminus (Figure 1A). The cassette also contained an mCherry reporter and a puromycin resistance gene for identification and selection of successfully integrated cells (Supplemental Figure 1A). The mEGFP fusion allows for visualization and quantification of total KCNQ1 in the cell, while the extracellular myc epitope can be used to visualize/quantify the population of cell surface KCNQ1 using anti-myc antibodies in non-permeabilized cells. Both the mEGFP fusion and the myc epitope insertion have been employed by other laboratories and have been shown not to impact KCNQ1 structure, function, or trafficking (46, 47). We generated a clonal stable cell line by integrating the cDNA for this construct, selecting for successfully integrated cells with puromycin antibiotic selection, sorting single cells into multi-well plates, and growing a clonal population from a single cell. Inducible expression of mycKCNQ1-mEGFP was confirmed by Western blot analysis (Supplemental Figure 1B). We note that KCNE1 was not present in this assay or in the flow cytometry trafficking assay (below). This is because the presence of KCNE1 blocks antibody recognition of the extracellular myc epitope inserted in the S1–S2 linker of KCNQ1 (after residue E146), which is required to quantify the population of KCNQ1 at the plasma membrane. The cryogenic electron microscopy (cryo-EM) structure of the KCNQ1-KCNE1 complex shows that KCNE1 makes contact with several residues in S1, including Q147, consistent with the notion that KCNE1 physically blocks the myc epitope (48).

We then used this stable cell line in an immunofluorescence-based trafficking assay compatible with high-content imaging of multi-well plates (Figure 1B). Expression of mycKCNQ1-mEGFP was induced 24 hours before cells were plated into 384-well plates. Each well was then treated with a screening compound (dissolved in DMSO) or DMSO (control) at a final concentration of 10 μM or 0.1%, respectively. Cells were treated with compound overnight (approximately 16 hours) to allow sufficient time for synthesis and trafficking of nascent KCNQ1 in the presence of compound but minimize off-target effects from longer treatment times, such as toxicity or degradation of the compound. After 16 hours, cells were fixed, stained, and imaged with a high-content imager. Cell surface KCNQ1, total KCNQ1, and the KCNQ1 trafficking ratio (surface fluorescence intensity/total fluorescence intensity) were quantified for each imaged cell, and average surface, total, and trafficking ratio values were calculated for each compound. Representative data from a screened plate are shown in Figure 1C.

To validate the assay, a clonal stable cell line expressing E115G mycKCNQ1-mEGFP, an LQT1-associated variant with known low total and cell surface levels of KCNQ1 (13), was used as a positive control. Consistent separation between negative control (DMSO-treated WT) cells and the positive control was observed (Supplemental Figure 1C). Cells were also tested to ensure that treatment with 0.1% DMSO did not alter mycKCNQ1-mEGFP surface, total, or trafficking values before screening (Supplemental Figure 1D).

**Figure 2:**
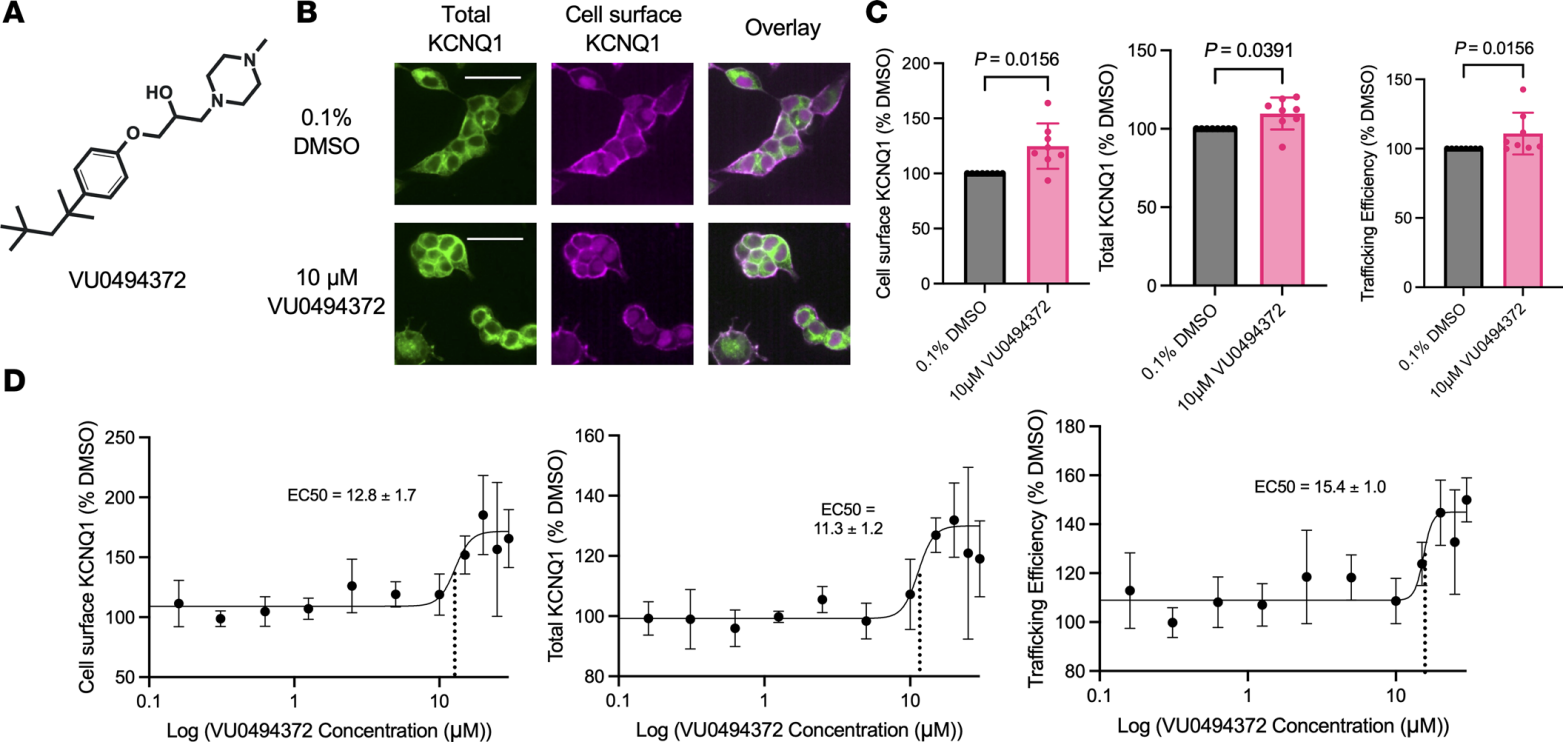
VU0494372 increases cell surface and total KCNQ1 in a dose-dependent fashion. (**A**) Chemical structure of VU0494372. (**B**) Images of total (left) or cell surface (middle) mycKCNQ1-mEGFP in LLP-int cells treated with either 0.1% DMSO (top) as a control or 10 μM VU0494372 (bottom). Scale bars: 40 μm; scale is the same in all images. (**C**) Expression and trafficking of mycKCNQ1 in stable cell line, as determined by flow cytometry–based trafficking assay. Values were normalized to 0.1% DMSO control within each replicate. *P* values were determined with Wilcoxon’s matched-pairs signed rank test. *N* = 8. Error bars represent standard deviation. (**D**) Dose-response curves for mycKCNQ1 cell surface levels (left), total levels (middle), and trafficking efficiency (right), in VU0494372-treated stable cell line. Values were normalized to 0.2% DMSO control in each replicate. The best-fit curve was generated and EC_50_ values were determined with GraphPad Prism. The curve in the “total” plot was constrained to have a maximum of 130%, and the curve in the “trafficking” plot was constrained to have a maximum of 145% owing to large variability at high concentrations. *N* = 3–5. Error bars represent standard deviation.

A total of 23,611 small molecules were screened from 5 libraries. Robust *z* scores (49–51) (Equation 1 in Methods) were calculated for cell surface KCNQ1, total KCNQ1, and KCNQ1 surface trafficking ratio for each screened compound on a plate-by-plate basis (Supplemental Figure 2). Compounds with a robust *z* score greater than 3 or less than –3 for any of the 3 quantified metrics (surface, total, trafficking ratio) that were not toxic were selected as initial hits (*n* = 386, 1.6% hit rate). A series of experiments were then performed to narrow the list of hits (Figure 1D). Hits were re-tested in triplicate, and 87 compounds with reproducible effects were identified. These hits were then counter-screened against an unrelated membrane protein, PMP22, to identify those that also altered PMP22 expression/trafficking, indicating nonspecific effects. Such nonspecific hits were excluded from further studies. Finally, for the top 21 hit compounds that met all desired criteria, a fresh supply of material was obtained from commercial sources. The new material was tested in a flow cytometry–based trafficking assay, which allowed for more precise quantification of protein levels and cell surface expression as well as calculation of a trafficking efficiency value (Equation 2 in Methods). Five compounds were found to have reproducible, specific effects (Supplemental Figure 3). Three of the top hits decreased cell surface and total KCNQ1 levels, and another increased total KCNQ1 dramatically but led only to a small increase in cell surface levels, leading to a net decrease in trafficking efficiency. The fifth hit, VU0494372 (Figure 2A), increased cell surface and total KCNQ1 levels as well as KCNQ1 trafficking efficiency in both the flow cytometry-based trafficking assay and in confocal microscopy (Figure 2B). We focus on VU0494372 in this study.

### *VU0494372 increases cell surface KCNQ1, total KCNQ1, and KCNQ1 trafficking efficiency in a dose-dependent manner*.

We first verified the effect of VU0494372 in the quantitative flow cytometry–based trafficking assay using a stable cell line expressing WT mycKCNQ1 (without mEGFP) to ensure that the effect of VU0494372 was not due to an interaction with mEGFP. We found that 10 μM VU0494372 treatment led to a 25% increase in cell surface KCNQ1, a 9.7% increase in total mycKCNQ1, and an 11% increase in mycKCNQ1 trafficking efficiency (Figure 2C). VU0494372 did not impact the unrelated membrane protein PMP22, indicating that the compound was not having a pleiotropic effect on membrane protein trafficking (Supplemental Figure 4). We also determined that VU0494372 exhibited a dose-dependent effect with a half-maximal effective concentration (EC__50__) of 12.8 ± 1.7 μM for increased cell surface KCNQ1 levels, an EC__50__ of 11.3 ± 1.2 μM for increased total KCNQ1 levels, and an EC__50__ of 15.4 ± 1.0 μM for increased KCNQ1 trafficking efficiency (Figure 2D). We also found that treating with 10 μM VU0494372 for longer (48 hours) led to an even larger effect on KCNQ1 surface levels, total levels, and trafficking (Supplemental Figure 5). Additional experiments, in which KCNQ1 expression was induced simultaneously with VU0494372 (or DMSO control treatment) exposure and KCNQ1 appearance at the plasma membrane was tracked, demonstrated that treatment with VU0494372 accelerated the appearance of KCNQ1 at the plasma membrane (Supplemental Figure 6), further supporting its role in increasing forward trafficking of KCNQ1. Together, these results demonstrate that VU0494372 increases cell surface levels, total levels, and trafficking efficiency of WT KCNQ1 in a treatment time– and dose-dependent manner, and increases forward trafficking of KCNQ1. We next sought to determine whether VU0494372 was also effective for disease-associated KCNQ1 variants.

### *Treatment with VU0494372 increases cell surface levels and trafficking efficiency of LQT1-associated variants*.

**Figure 3:**
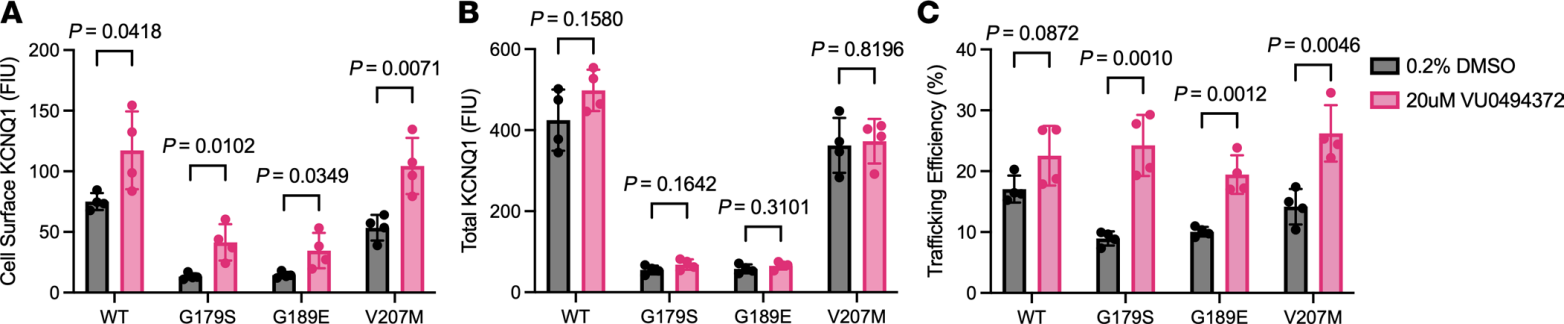
Treatment with VU0494372 increases cell surface levels and trafficking efficiency of LQT1-associated variants. Cell surface (**A**), total (**B**), and trafficking efficiency (**C**) data for WT, G179S, G189E, and V207M mycKCNQ1 in “population” stable cell lines, as determined by flow cytometry–based trafficking assays, after treatment with 0.2% DMSO or 20 μM VU0494372 for 16 hours. *N* = 4. Error bars represent standard deviation, and *P* values were determined with 2-tailed unpaired *t* tests.

We next determined the effect of VU0494372 on the surface levels, total levels, and trafficking efficiency of LQT1-associated KCNQ1 variants. We selected 3 previously characterized variants: G179S and G189E, known to be severely dysfunctional in trafficking and function, and V207M, known to have a milder, but still disease-causing, phenotype (12, 13). We generated “population” stable cell lines with WT mycKCNQ1 and the three LQT1-associated variants similarly to our approach for the original stable cell lines but omitting the final clonal population growth step. Cells were treated with 20 μM VU0494372 (a concentration above the determined EC__50__, to ensure maximal effect size) for 16 hours. We found that treatment with 20 μM VU0494372 resulted in significantly increased cell surface KCNQ1 and KCNQ1 trafficking efficiency for all three LQT1 variants (Figure 3, A and C). The variants exhibited a greater response to VU0494372 compared with the 1.5-fold increase for WT KCNQ1. G189E and G179S exhibited greater than 2-fold and greater than 3-fold increases in cell surface levels, respectively, while V207M exhibited an approximately 2-fold increase. Increases in trafficking efficiency represented a similar trend, with all 3 variants exhibiting a larger-magnitude change in trafficking than WT after treatment with VU0494372. Total levels of WT KCNQ1 increased slightly, but the change was not statistically significant, likely because of increased variability in the population stable cell line compared with the clonal line used previously. Total levels of the 3 tested variants did not change (Figure 3B). These results demonstrate that these three LQT1-associated KCNQ1 variants are responsive to VU0494372 treatment and exhibit even larger increases in surface and trafficking values than WT. After determining the effects of VU0494372 on WT and variant KCNQ1, we investigated its mechanism of action.

### *VU0494372 does not alter KCNQ1 transcription, degradation, or stability*.

**Figure 4:**
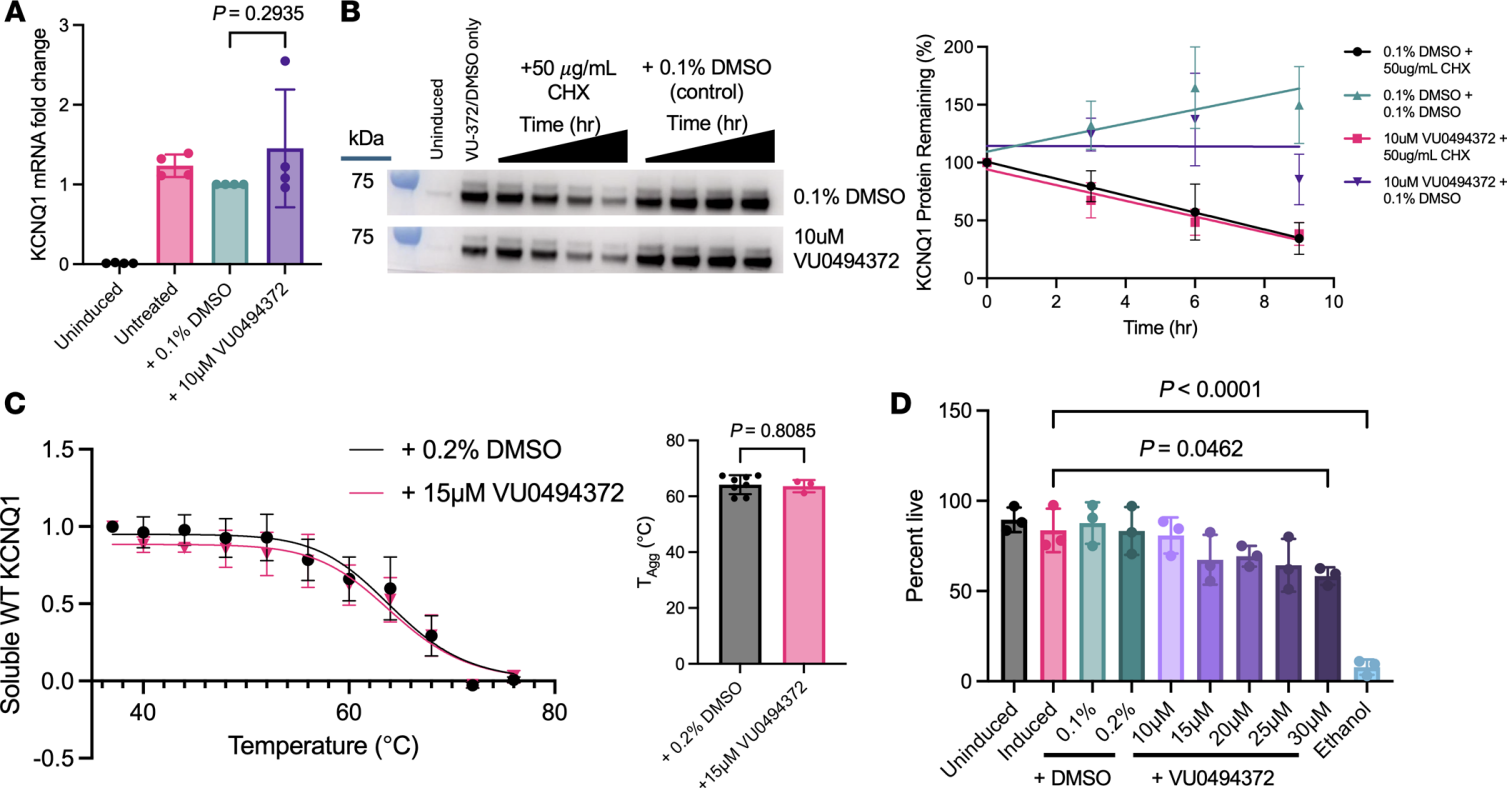
VU0494372 effects do not involve modulation of KCNQ1 degradation, transcription, thermal stability, or cell toxicity. (**A**) Fold change in KCNQ1 mRNA levels, as determined by qPCR, relative to samples treated with 0.1% DMSO. *N* = 4. *P* values were calculated with Kruskal-Wallis tests with Dunn’s multiple-comparison test as follow-up. Error bars represent standard deviation. (**B**) Cycloheximide chase assay to determine the rate of KCNQ1 degradation. Left: Remaining KCNQ1 was detected with Western blotting. Representative full blots are shown in Supplemental Figure 7. Right: KCNQ1 band intensities were normalized to β-actin loading control bands and plotted as a percentage remaining from the 0-hour time point. *N* = 3 replicates, for which error bars represent standard deviation. Lines of best fit were generated with GraphPad Prism. (**C**) Left: Thermal aggregation curve from cellular thermal shift assay (CETSA) experiments. Bands corresponding to KCNQ1 were quantified and T_agg_ values were calculated for *N* = 3 (VU0494372-treated) or 5 (DMSO-treated) replicates. Curves of best fit were modeled with GraphPad Prism. Right: T_agg_ quantifications were calculated based on the inflection point of the modeled curves in each individual CETSA replicate. A 2-tailed unpaired *t* test was used to determine the *P* value. Error bars on both graphs represent standard deviations. Full representative blots are shown in Supplemental Figure 7. (**D**) Toxicity of 0.1%–0.2% DMSO or 10–30 μM VU0494372, determined by using trypan blue to assess cell viability. One-way ANOVA with Dunnett’s multiple-comparison follow-up test was used to determine *P* values. *N* = 3. Error bars represent standard deviation.

To investigate the mechanism of action, we conducted a series of studies testing the impact of VU0494372 on KCNQ1 expression, degradation, and stability. Because treatment with VU0494372 increased total levels of KCNQ1, we investigated whether this change was due to increased KCNQ1 production or a reduction in KCNQ1 degradation. We began by examining whether the increased levels of KCNQ1 in cells were a result of VU0494372 altering KCNQ1 at the transcriptional level. We used quantitative reverse transcription PCR (RT-qPCR) to quantify KCNQ1 mRNA levels in our stable cell line and found that treatment with VU0494372 did not alter the levels of KCNQ1 mRNA (Figure 4A). We next performed cycloheximide chase assays, which involve inhibiting synthesis of new protein and tracking target protein degradation over time, to determine the effect of VU0494372 on the rate of degradation of KCNQ1. These experiments determined that the cellular half-life of WT KCNQ1 was approximately 7 hours, and that treatment with VU0494372 did not change the rate of KCNQ1 degradation (Figure 4B). Because the overall efficiency of trafficking of KCNQ1 was increased, we sought to determine whether this was mediated by an alteration in the stability of KCNQ1, which could facilitate proper folding and trafficking. A cellular thermal shift assay (CETSA) was used to calculate a thermal aggregation temperature (T__agg__) for KCNQ1 under cellular conditions with and without 15 μM VU0494372. Treatment with VU0494372 did not alter the thermal stability of KCNQ1 (Figure 4C).

Finally, to rule out toxicity-based mechanisms (e.g., apoptosis) as a confounding factor in the effect of VU0494372, we tested whether VU0494372 induced cellular toxicity. Cells were treated with 10–30 μM VU0494372 for 16 hours followed by assessment of viability using trypan blue staining. The compound did not significantly impact the viability of the mycKCNQ1-expressing stable cell line at concentrations of up to 25 μM (Figure 4D).

Together, these experiments rule out alterations in transcription, degradation, or global stability as possible mechanisms of action for VU0494372 and show that VU0494372 is not significantly toxic to cells at the concentrations used. We next investigated whether VU0494372 impacted the function of KCNQ1, which could provide further insight into its mechanism of action.

### *Functional effects of acute and chronic VU0494372 exposure*.

**Figure 5:**
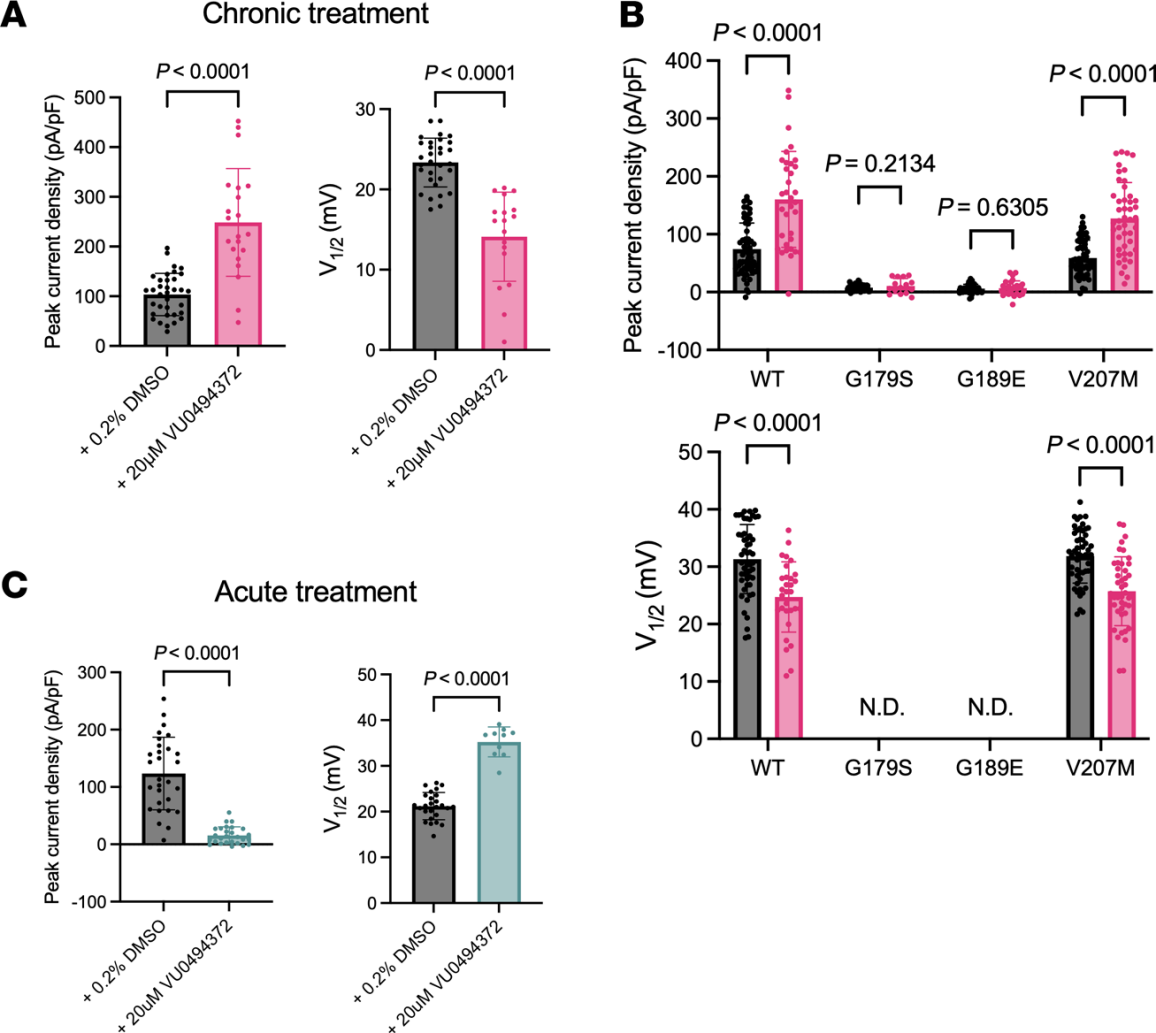
Treatment with VU0494372 leads to an increase in WT and V207M KCNQ1-KCNE1 activity. (**A**–**C**) KCNQ1 channel function quantified in CHO-K1 cells stably expressing KCNE1 (CHO-KCNE1) and transfected with WT or variant KCNQ1 using automated patch clamp electrophysiology (*n* = 10–60 cells per group). Error bars represent standard deviation, and *P* values were determined with 2-tailed unpaired *t* tests. (**A**) Peak current density (left) recorded 1,900 milliseconds after the start of the voltage pulse, and V_1/2_ of activation (right) of WT KCNQ1 after 16-hour (chronic) treatment with 0.2% DMSO or 20 μM VU0494372. Compound was removed before recording (*n* = 18–35 cells were quantified from 2 separate experimental replicates). (**B**) Peak channel current density (top) and V_1/2_ of activation (bottom) for CHO-KCNE1 cells transiently transfected with WT or LQT1 variant G179S, G189E, or V207M KCNQ1 after chronic treatment with 0.2% DMSO or 20 μM VU0494372. Compound or DMSO was washed off before recording (*n* = 15–60 cells per group). N.D., not determined, due to low current amplitude. (**C**) Peak current density (left) and V_1/2_ of activation (right), as determined for CHO-KCNE1 cells transfected with WT KCNQ1. Cells were treated with 0.2% DMSO or 20 μM VU0494372 for 5 minutes (acute treatment) before recording, and treatment remained present during recording (*n* = 10–29). Average whole current traces for all electrophysiology experiments are shown in Supplemental Figure 8.

After determining that VU0494372 increases cell surface levels of WT and LQT1-associated KCNQ1 variants, we investigated whether VU0494372 treatment led to corresponding changes in I__Ks__ (KCNQ1 + KCNE1 current). Automated whole-cell patch clamp electrophysiology experiments were conducted with CHO cells stably expressing KCNE1 (CHO-KCNE1) and transiently transfected with WT KCNQ1. Average whole current traces for all electrophysiology experiments are shown in Supplemental Figure 8. First, cells were treated with 20 μM VU0494372 for 16 hours (chronic treatment) to ensure maximal effect size and allow time for alterations in cell surface levels to occur. VU0494372 was removed before recording of currents. We found that WT KCNQ1-KCNE1 peak current density significantly increased after treatment with VU0494372, and the voltage of half-maximal activation (V__1/2__) of activation of KCNQ1 shifted significantly to less depolarized potentials after treatment (Figure 5A). These effects were dose dependent, although full dose-response curves could not be obtained owing to toxicity at concentrations greater than 20 μM (Supplemental Figure 9). The increase in peak current density likely reflects an increase in the number of channels at the cell surface, and the shift in the V__1/2__ of activation of KCNQ1 reflects a change in the gating of KCNQ1, which could be occurring through any one of several possible mechanisms (see Discussion).

We next determined whether VU0494372 alters the function of disease-associated variants of KCNQ1. CHO-KCNE1 cells transiently transfected with WT, G179S, G189E, or V207M KCNQ1 were treated with 20 μM VU0494372 for 16 hours, followed by removal of the compound. Automated whole-cell patch clamp experiments demonstrated that chronic treatment of V207M KCNQ1 led to significant increases in current and depolarization of activation V__1/2__ similarly to WT. However, G179S and G189E did not exhibit changes in KCNQ1 current density, and their V__1/2__ of activation could not be determined because of low overall current (Figure 5B). This suggests that even though surface trafficking of these variants is very significantly enhanced, the surface-trafficked population of the channel remains dysfunctional.

Automated whole-cell patch clamp studies of CHO-KCNE1 cells transiently transfected with WT KCNQ1 and treated with 0.2% DMSO or 20 μM VU0494372 for 5 minutes (acute exposure, no washing) were also conducted. For this experiment, VU0494372 or DMSO (control) was added 5 minutes before whole-cell recordings and maintained throughout the experiment. These experiments showed an effect opposite to that of chronic treatment — a decrease in KCNQ1 peak current density and a positive shift in V__1/2__ of activation (Figure 5C). These changes were consistent with direct inhibition by VU0494372 of KCNQ1 channel function. The decrease in KCNQ1 currents after this short-term exposure strongly suggests that the small molecule is interacting directly with KCNQ1 to reduce channel activity, while the shift in the activation V__1/2__ reflects alteration of the voltage sensitivity of gating.

Together, our electrophysiological studies demonstrate that chronic treatment with VU0494372, followed by compound removal, leads to increases in WT and V207M KCNQ1 function, consistent with increased trafficking to the plasma membrane. On the other hand, acute continuous exposure leads to decreases in WT KCNQ1 function, indicating that VU0494372 can act as a channel blocker while increasing trafficking efficiency. This led us to next investigate whether VU0494372 is directly interacting with KCNQ1.

### *Competition assays support direct interaction between VU0494372 and KCNQ1*.

**Figure 6.**
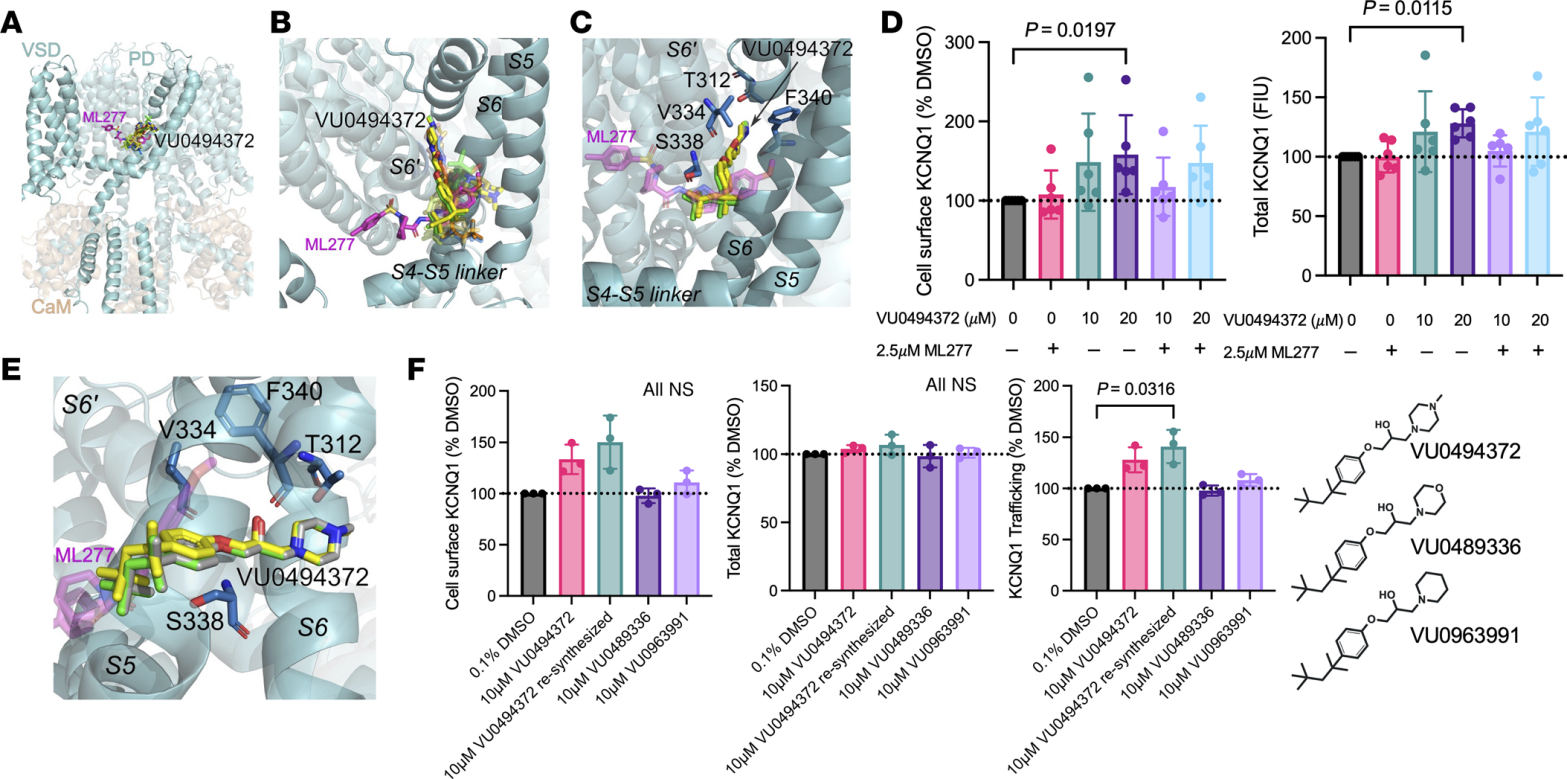
Computational docking, electrophysiology data, and competition assays provide support for possible direct interaction between VU0494372 and KCNQ1. (**A**) Overlay of PDB 7XNK showing ML277 (magenta) bound to voltage sensor–active, pore-open KCNQ1 and the top 10 docked poses of VU0494372 (assorted colors) ranked by RosettaLigand interface score. VSD, voltage-sensing domain; PD, pore domain; CaM, calmodulin. (**B**) Zoomed and rotated view of **A**. Docked VU0494372 poses overlap with bound ML277 in KCNQ1. Labeled residues line the predicted pocket and interact with VU0494372. (**C**) Four poses from **B** of the most frequently observed orientation of VU0494372 docked to KCNQ1. (**D**) Competition between ML277 and VU0494372 as determined with a flow cytometry–based trafficking assay. Cell surface (left) and total (right) mycKCNQ1 levels in LLP-int cells were determined with and without ML277 and VU0494372. *N* = 6. *P* values were determined with Kruskal-Wallis non-parametric test with Dunn’s multiple-comparison test for follow-up. (**E**) Rotated and zoomed view of **C** to illustrate the orientation of VU0494372. (**F**) Cell surface levels (left), total levels (middle), and trafficking efficiency (right) of WT KCNQ1 in an LLP-int WT KCNQ1–expressing cell line after treatment with 10 μM VU0494372, VU0489336, or VU0963991 (or 0.1% DMSO as a control) for 16 hours, quantified with a flow cytometry–based trafficking assay. *N* = 3. Compound structures are shown on the far right. *P* values were determined with Kruskal-Wallis non-parametric test with Dunn’s multiple-comparison test for follow-up.

The observation that acute, continuous exposure of KCNQ1-expressing CHO-KCNE1 cells to VU0494372 inhibits channel function suggests that this compound directly interacts with KCNQ1. To strengthen evidence for direct interaction, we used a combination of experimental and computational molecular modeling approaches to predict and evaluate potential binding sites of VU0494372 to KCNQ1. VU0494372 was blind-docked to 2 different cryo-EM structures of KCNQ1, representing 2 different conformations of the protein: a voltage sensor up with pore closed conformation (Protein Data Bank [PDB] 8SIK) (52), and a voltage sensor active state with pore open conformation (PDB 7XNK) (53). We used DiffDock (54) to generate predicted binding poses. We then refined and scored the DiffDock poses with the RosettaLigand (55, 56) small-molecule docking protocol for high-resolution refinement. The best-scoring poses with the most favorable binding energies positioned VU0494372 proximal to the KCNQ1 pore domain, forming contacts with the S6 transmembrane helix and the S5–S6 linker (Figure 6, A–C).

All docked poses partially overlapped with the binding site of a known KCNQ1 activator, ML277 (41) (Figure 6, A–C). ML277 has an EC__50__ of 0.26 μM and does not alter the trafficking of KCNQ1, even at concentrations as high as 10 times the EC__50__ (41) (Figure 6D). We therefore investigated whether ML227 and VU0494372 compete for the same binding site. We treated LLP-int mycKCNQ1 cells with 2.5 μM ML277 and either 10 μM or 20 μM VU0494372 and used our flow cytometry trafficking assay to quantify cell surface and total KCNQ1. We then compared these values with those from ML277 or VU0494372 alone and found that cotreating with ML277 reduced the effects of VU0494372 on KCNQ1 levels (Figure 6D), consistent with the notion that the two small molecules are competing for the same binding site.

We also sought to determine whether alterations to the chemical substituents of VU0494372 predicted to interact with KCNQ1 impacted its effect on the channel, which could indicate altered binding. One end of the compound, containing a methylpiperazine ring, was predicted to penetrate into KCNQ1 and interact with several residues in the KCNQ1 pore domain (Figure 6E). The Vanderbilt Molecular Design and Synthesis Core synthesized VU0494372 and two analogs (VU0489336 and VU0963991) with modifications to this methylpiperazine ring present in the parent compound (Figure 6F). These two analogs exhibited reduced effects on cell surface KCNQ1 and trafficking efficiency (Figure 6F), suggesting that this portion of the compound was essential for maximal effect of VU0494372 on surface trafficking, possibly because of direct interaction with KCNQ1 residues in the pore domain.

Combined, these data suggest that VU0494372 interacts directly with KCNQ1 near the ML277 binding site with the methylpiperazine ring oriented toward the channel pore, potentially interacting with residues including T312, V334, and F340 in the S6 helix. Whether VU0494372 occupies all 4 sites in the KCNQ1 homotetramer is not established by our data, but seems likely. Also not established by our data is the possibility for cooperativity of binding.

## Discussion

### *A versatile high-throughput screening discovery assay for compounds that alter KCNQ1 levels and/or trafficking*.

In this work, we used a high-content image-based high-throughput screening (HTS) trafficking assay to test the hypothesis that KCNQ1 expression and/or trafficking can be altered with small molecules, a result that provides proof-of-concept support for the potential use of small molecules of this class to remedy KCNQ1 dysfunction in LQT1. We screened nearly 24,000 small molecules for their impact on WT KCNQ1 cell surface levels, total levels, and trafficking efficiency. This approach is advantageous because it enables identification of compounds that target a single aspect of KCNQ1 expression and trafficking, such as changing surface levels without altering total levels. This assay is additionally useful because it is bidirectional; it can be used to identify small molecules that increase KCNQ1 levels and trafficking and are relevant to LQTS as well as small molecules that lower KCNQ1 levels and/or trafficking efficiency. In fact, 3 of the top 5 hit compounds decreased surface and total KCNQ1 levels (Supplemental Figure 3). Compounds of this latter class are of interest as potential drug leads for rare cardiac disorders such as short QT syndrome and familial atrial fibrillation, where the disease is caused by excessive KCNQ1 expression and/or trafficking or by unregulated KCNQ1 channel function (57, 58). This assay may also be adapted for use with more complex systems such as cardiomyocytes or organoids.

### *VU0494372 enhances both total and surface levels of KCNQ1 and increases its surface trafficking efficiency*.

VU0494372 is a small molecule identified by our HTS screen that, after 16 hours of 20 μM (nearly twice EC__50__) treatment, increased WT KCNQ1 cell surface levels by about 40% (Figures 2 and 3). Even larger effect sizes were observed with longer treatment times (Supplemental Figure 5), and impacts on initial forward trafficking were also confirmed (Supplemental Figure 6). Moreover, treatment with 20 μM VU0494372 for 16 hours led to enhancements of 2-fold and higher of cell surface levels and trafficking efficiency for the disease-linked variants G179S, G189E, and V207M (Figure 3). These effect sizes were larger than those observed for WT KCNQ1, suggesting that disease-associated variants in KCNQ1 may be more sensitive to the compound than WT. Additional results ruled out several possible artifactual explanations for how VU0494372 enhances KCNQ1 trafficking and indicate that VU0494372 specifically targets KCNQ1, appearing to bind directly (Figure 6). Additionally, our studies found that VU0494372 does not change the rate of KCNQ1 degradation nor broadly alter KCNQ1’s thermal stability in cells (Figure 4). These experiments demonstrate that VU0494372 likely acts by increasing the forward trafficking of KCNQ1.

While a number of KCNQ1 activators and blockers have previously been identified (41–44, 59, 60), VU0494372 is the first small molecule that increases cell surface levels of KCNQ1. These results support our hypothesis that KCNQ1 expression and trafficking can be modified by small molecules and suggest that pharmacological enhancement of KCNQ1 trafficking is a potential route for treating LQT1. It is particularly encouraging both that the studied disease-associated KCNQ1 variants exhibit larger improvements in surface trafficking than WT and also that all three tested variants were responsive to VU0494372. It is estimated that about half of the hundreds of known LQT1-associated KCNQ1 variants exhibit impaired cell surface expression (12, 13), so a small-molecule trafficking corrector that remedies these variants would be broadly useful for treating LQT1.

### *VU0494372 increases WT I__Ks__ after chronic treatment*.

Our functional studies show that treatment of KCNQ1/KCNE1-coexpressing CHO cells with VU0494372 for 16 hours followed by compound removal results in increased I__Ks__ (Figure 5). However, a depolarizing shift in the activation V__1/2__ of the I__Ks__ channel was also observed (Figure 5A). This change in the voltage sensitivity of channel gating could be the result of any one of several mechanisms, including either drug-induced alteration of the phosphorylation of the channel or stabilization of an interaction with a chaperone or accessory protein, both of which have previously been shown to shift the V__1/2__ of activation for KCNQ1 (61–63). Overall, these changes in V__1/2__ also represent a potentiating effect, though the mechanism is not yet clear. A similar effect has been observed for the KCNQ2-activating drug retigabine (64, 65).

Additionally, the effect of this compound in CHO cells coexpressing KCNE1 and KCNQ1 confirms that VU0494372 still exerts its effect on KCNQ1 in the presence of its accessory protein KCNE1, despite initially being identified in HTS screens conducted in the absence of KCNE1. Effectiveness on both KCNQ1 alone and KCNQ1-KCNE1 is not exhibited by some modulators of KCNQ1. For example, ML277’s and R-L3’s effects on KCNQ1 function are hindered in the presence of KCNE1 (43, 66). Since the KCNQ1-KCNE1 complex is the active form of the channel in the heart (63), this result provides additional evidence that small molecules such as VU0494372 have potential for clinical use and benefit.

### *Variant-specific dependence of the impact of VU0494372 on channel function*.

While VU0494372 increased the cell surface levels and trafficking efficiency of three LQT1-associated variants, G179S, G189E, and V207M (Figure 3), only V207M showed significant increases in channel conductance after treatment with VU0494372 (Figure 5B), despite the fact that cell surface levels and trafficking efficiency improved 2-fold or more for all 3 studied variants. This can likely be explained by the degree of dysfunction of each variant, which has been previously characterized (12, 13). G179S and G189E are known to cause severe dysfunction of KCNQ1. We have previously demonstrated not only that both are destabilized and have poor cell surface trafficking, but that they also exhibit extremely low KCNQ1 conductance (12, 13). By contrast, V207M exhibits a less severe phenotype with only modest impairments in trafficking, function, and stability (13).

Our results suggest that some “milder” disease-associated variants, like V207M, that mistraffic but are still able to function may be susceptible to rescue by compounds that modulate expression/trafficking. Multi-pronged approaches like treating with both a trafficking corrector and a channel activator may be needed to remedy more severely dysfunctional variants such as G179S and G189E. This dual approach has been used for variants in CFTR that cause cystic fibrosis, in particular the common ΔF508 form, which causes both mistrafficking and loss of function of the channel (35–37, 67).

### *Interaction of VU0494372 with KCNQ1*.

Our data suggest direct interaction between KCNQ1 and VU0494372 as the basis for mediating its mechanism of action, as functional testing in the presence of VU0494372 demonstrated an inhibitory effect (Figure 5C). Computational docking of VU0494372 to KCNQ1 suggested a favorable binding site between the voltage-sensing and pore domains of KCNQ1 with the methylpiperazine ring of VU0494372 making contacts with pore domain residues (Figure 6, A–C and E). Subsequent competition experiments with the KCNQ1 activator ML277 (53, 68), which is known to bind near this site, and examination of VU0494372 analogs with modifications to the methylpiperazine ring that further reduced activity supported the potential for direct interaction (Figure 6, D and F).

It cannot be ruled out that the apparent competitive effects of VU0494372 and ML277 are seen for a reason other than direct competition, such as allostery. Our results also do not inform regarding the stoichiometry of VU0494372 binding to the KCNQ1 homotetramer, or regarding possible cooperativity of binding of this compound to multiple sites. Further experiments are required to address these issues.

### *Limitations of VU0494372*.

While the ability of VU0494372 to enhance cell surface trafficking of KCNQ1 represents an encouraging development, this compound does exhibit some limitations as a potential lead for small-molecule drug discovery. An addressable limitation is that the EC__50__ of the effect of VU0494372 on KCNQ1 trafficking is in the range of 10–15 μM, higher than is desirable for a viable drug candidate. It is possible that the potency of this compound can be improved by iterative medicinal chemistry and analog testing. A modest first step toward these structure activity relationship studies is illustrated by data in Figure 6F.

A more serious limitation is the fact that VU0494372 acts as a channel blocker (Figure 5C), albeit reversibly so (Figure 5A). This phenomenon has been previously observed with some of the original “pharmacological chaperones,” which increased surface trafficking of several different G protein–coupled receptors, even though they were known receptor antagonists (33, 69). Small molecule–induced increases in trafficking combined with acute channel block have also been observed for potassium channel hERG (K__V__11.1), variants of which cause type 2 long QT syndrome (5). Several drugs, including astemizole (an antihistamine), dofetilide (an approved anti-arrhythmia drug), and E4031 (an investigational anti-arrhythmia drug), directly bind hERG, increase its trafficking, and also block channel function (70), likely by binding to a site important for channel function or by blocking the channel pore (70). The KCNQ1 channel inhibitory property of VU0494372 would likely need to be eliminated through SAR before a compound derived from VU0494372 could be used to treat LQT1. Nevertheless, identification of VU0494372 as a corrector of KCNQ1 trafficking is encouraging for future LQT1 drug discovery efforts.

### *Future directions*.

Much work remains to optimize VU0494372 and evaluate its mechanism of action. Future experiments can be conducted to confirm the hypothesized location of VU0494372 binding and inform regarding the stoichiometry of binding. Additionally, testing the impact of VU0494372 on a larger number of KCNQ1 variants will inform regarding the degree to which it acts to globally correct the trafficking defects of variants distributed across the multiple domains of KCNQ1. Lastly, experiments using heterozygous conditions and more physiologically relevant models, like induced pluripotent stem cell–derived cardiomyocytes (IPSC-CMs), could inform regarding the likelihood that VU0494372 would be effective in cardiac tissue under physiological conditions. While much of the protein trafficking machinery is universal to all cells, cardiomyocytes have unique cellular structures (such as T tubules) that may impact the trafficking pathways of proteins like KCNQ1 (71–73). In this regard, it is encouraging that we and other groups have shown that results in model cell lines can sometimes be replicated in cardiomyocyte models (12, 72).

## Methods

Some methods presented below are summaries, with a more detailed version presented in Supplemental Methods.

### Sex as a biological variable section

Sex was not considered as a biological variable, as no human and/or animal models were used in these studies.

### Cell culture conditions

All mammalian cells were cultured at 37°C and 5% CO__2__. HEK293T “LLP-int” cells (shared with us by Kenneth Matreyek, Case Western Reserve University, Cleveland, Ohio, USA) and all stable cell lines generated from LLP-int cells were cultured in high-glucose DMEM without sodium pyruvate (Gibco) supplemented with 100 U/mL penicillin/streptomycin (P/S; Gibco) and 10% tetracycline-negative FBS (Corning) and grown in flasks coated with poly-l-lysine (PLL; Millipore). HEK293 cells (American Type Culture Collection [ATCC]) were cultured in high-glucose DMEM without sodium pyruvate (Gibco) with P/S and FBS. CHO-K1 cells (ATCC) constitutively expressing human KCNE1 (designated CHO-KCNE1 cells) used for electrophysiology experiments were generated as previously described (74) using the Flp-In system (Thermo Fisher Scientific) and were grown in F-12 Ham nutrient medium (Gibco/Invitrogen) supplemented with 10% FBS (Atlanta Biologicals) and P/S and maintained under selection with hygromycin B (600 μg/mL).

### KCNQ1 constructs and mutagenesis

All experiments were conducted with “mycKCNQ1”: full-length human KCNQ1 (GenBank accession number AF000571) with a myc epitope (EQKLISEEDL) inserted after residue E146 in the S1–S2 extracellular loop of the KCNQ1 voltage-sensing domain (46); or “mycKCNQ1-mEGFP”: mycKCNQ1 with a monomeric enhanced green fluorescent protein (mEGFP; containing the A206K mutation) fused to the C-terminus. Fusion protein generation is described in detail in Supplemental Methods, and primers used are listed in Supplemental Table 1.

### Landing pad cells

Stable cell lines expressing mycKCNQ1-mEGFP WT, mycKCNQ1-mEGFP E115G, and mycKCNQ1 WT and G179S, G189E, and V207M mycKCNQ1 used for high-throughput screening (HTS) and subsequent experiments were generated using LLP-int HEK293T cells, described in detail in previous publications (45, 75), which contain a tetracycline-inducible genomic “landing pad” DNA integration site and a Bxb1 integrase gene. Target DNA flanked by a complementary integration sequence in a promoterless “shuttle vector” can be incorporated at this site. Details of stable cell line generation are described in Supplemental Methods.

### Imaging-based high-throughput screen

To screen for modifiers of KCNQ1 trafficking, expression of WT mycKCNQ1-mEGFP (used for compound testing) or E115G mycKCNQ1-mEGFP (used as a control) was induced by treatment of the respective stable cell line with 1 μg/mL doxycycline 24 hours before plating of cells into 384-well microscopy plates (Greiner, 781090) coated with PLL. Compounds used for HTS were obtained from the Vanderbilt High Throughput Screening Core Facility dissolved in DMSO at 10 mM. Four to six hours after plating, compounds were diluted in media with doxycycline and added to wells to a final concentration of 10 μM (0.1% DMSO). 0.1% DMSO was used as a control. Cells were incubated with compounds for about 16 hours before staining. The population of mycKCNQ1-mEGFP at the cell surface was labeled by incubation of cells with an anti-myc mouse antibody (Cell Signaling, 2276S) diluted 1:500 in medium without P/S and FBS for 30 minutes at room temperature. Cells were then fixed with 4% paraformaldehyde (Santa Cruz Biotechnology) for 30 minutes. After fixing, cells were washed 3 times with phosphate-buffered saline with calcium and magnesium (PBS++) (MilliporeSigma). Cells were then incubated with an anti-mouse Alexa Fluor 647–conjugated secondary antibody (Cell Signaling, 4410S) diluted 1:1,000 in antibody dilution buffer (PBS++ with 5% goat serum [Gibco]) for 45 minutes. Cells were again washed 3 times with PBS++, then permeabilized with PBS++ with 0.3% Triton X and 0.1% bovine serum albumin (BSA) for 15 minutes. Cell membranes were then stained with CF405S-conjugated wheat germ agglutinin (Biotium, 29027) at a 1:200 dilution in PBS++ for 30–45 minutes. Lastly, cells were washed 3 times with PBS++ and imaged using an ImageXpress Micro Confocal high-content imager (Molecular Devices) (Supplemental Methods).

### Compounds screened

All screened small molecules were purchased from commercial sources, stored frozen in DMSO at a concentration of 10 mM, and managed by the Vanderbilt High Throughput Screening Facility. Small molecules from the following libraries were screened: (a) FDA-approved Drug Library (Selleck Chemicals); (b) Vanderbilt Discovery Collection (purchased from Life Chemicals), a curated list of about 100,000 compounds selected by Vanderbilt scientists to represent maximum chemical diversity and minimal pan-assay interference — the first 20,000 compounds of this library, which represent the chemical diversity of the full ~100,000 compounds in the library, were screened in this work; (c) Bio-Active Lipid library (Cayman Chemical); (d) Steroid-like Compounds library (ChemDiv).

After initial screening, fresh VU0494372 (product F1562-0024) powder was purchased from Life Chemicals and was used for all subsequent experiments.

### Hit picking and quality control

Data obtained from the custom analysis module were visualized and analyzed using the Vanderbilt High Throughput Screening Core Facility’s data visualization system Waveguide in conjunction with BIOVIA Pipeline Pilot (Dassault Systemes). Robust *z* score (a median-based modified *z* score) (49–51) was calculated for each compound with the following equation:
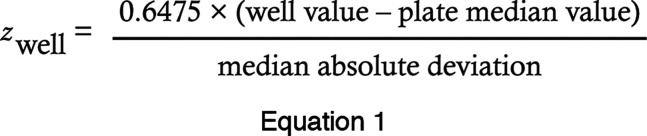


Hits were defined as compounds with a robust *z* score greater than 3 or less than –3. Additional quality control metrics (cell count, laser focus score, *z*′, and percent coefficient of variance, described in Supplemental Methods) were calculated for each plate, to ensure the robustness of the screen and quality of each individual plate.

During hit confirmation experiments, the same values were calculated for each compound for each biological replicate. Hits were considered confirmed if they met the *z* score threshold of greater than 3 or less than –3 in at least 2 of the 3 biological replicates.

### Flow cytometry–based trafficking assay

Trafficking of KCNQ1 in the LLP-int WT mycKCNQ1 cell line was quantified using a previously published flow cytometry–based trafficking assay (13, 21). In a typical experiment, KCNQ1 expression was induced for approximately 24 hours, cells were treated with compound or DMSO control for approximately 16 hours, and then cell surface and internal populations of KCNQ1 were stained. In some experiments, induction and/or treatment time were varied. Cells were washed once with PBS (Corning) plus 0.2% sodium azide (PBS-FC), detached with a solution of PBS-FC plus 0.5 mM EDTA and 0.5% BSA (Fisher Bioreagents), and pelleted by centrifugation. Cell surface mycKCNQ1 was stained by incubation of cells in PBS-FC plus 5% FBS (PBS-FC + FBS) containing anti-myc antibody conjugated to phycoerythrin (PE) (Cell Signaling, 3739S) or Alexa Fluor 488 (Cell Signaling, 2279S) at a 1:100 dilution for 30 minutes. Cells were then fixed by addition of Fix & Perm Medium A (Invitrogen, GAS004) for 15 minutes. Cells were washed twice, then permeabilized for 30 minutes while internal mycKCNQ1 was simultaneously labeled with Fix & Perm Medium B (Invitrogen, GAS004) with anti-myc Alexa Fluor 647–conjugated antibody (Cell Signaling, 2233S) diluted 1:100. Cells were again washed 2 times and then resuspended in PBS-FC + FBS for flow cytometry. The following controls were also prepared for use in flow cytometry analysis: untransfected (or uninduced) and unstained cells, single-color controls (total protein stained with one antibody), and background staining control (uninduced or empty vector–transfected cells stained with antibodies).

### Flow cytometry

Flow cytometry analysis was conducted using a 5-laser BD LSRFortessa flow cytometer (BD Biosciences). Compensation was determined using unstained cells and single-color controls described above. Gates were drawn to select single cells that were mCherry positive (for LLP-int cell lines) or EGFP positive (for transiently transfected cells). Alexa Fluor 647 and Alexa Fluor 488 (LLP-int cell lines) or PE (transiently transfected cells) fluorescence intensities were then quantified for 2,500 cells per sample. Single-cell data were exported using FlowJo data analysis software and used to calculate average cell surface KCNQ1, total KCNQ1, and KCNQ1 trafficking efficiency. The trafficking efficiency was calculated with the following equation:
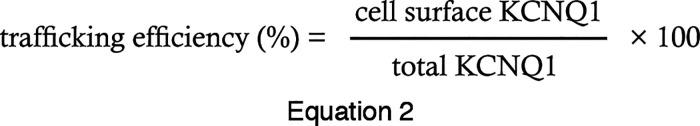


Details of background subtraction and brightness correction calculations for cell surface KCNQ1 and total KCNQ1 are described in Supplemental Methods.

### RNA extraction, cDNA generation, and qPCR

Relative KCNQ1 and GAPDH (control) mRNA levels were determined using quantitative reverse transcription PCR (RT-qPCR). Expression of mycKCNQ1 in the LLP-int WT mycKCNQ1 cell line was induced by treatment with 1 μg/mL doxycycline for 24 hours, and then cells were treated with 0.1% DMSO or 10 μM VU0494372 for 16 hours. RNA was extracted and purified from cells as previously described (76) (Supplemental Methods). RNA concentration and A260/A280 ratio were determined with a Thermo Fisher Scientific NanoDrop Lite Spectrophotometer. RNA was then reverse-transcribed into cDNA using a SuperScript IV VILO Master Mix kit (Invitrogen, 11766050). For qPCR experiments, diluted cDNA and qPCR primers were combined with Fast SYBR qPCR Master Mix (Applied Biosystems, 4385612) and plated in triplicate in a qPCR clear reaction plate (Applied Biosystems, A36924). Details of qPCR primers, thermal cycling protocol, and calculation of fold change are described in detail in Supplemental Methods.

### Cycloheximide chase assay

WT mycKCNQ1 expression was induced in LLP-int cells by addition of doxycycline to the medium 24 hours before additional treatment with either 0.1% DMSO or 10 μM VU0494372 for 16 hours. To assess the rate of KCNQ1 degradation, cells were then treated with either 50 μg/mL cycloheximide (Cell Signaling, 2112S) or 0.1% DMSO (as a control) for 0, 3, 6, or 9 hours. Soluble KCNQ1 was collected using the same lysis method as in CETSA experiments. Total protein in lysates was quantified using Pierce 660 nm protein assay reagent (Thermo Fisher Scientific, 22660), and 6–7 μg protein was loaded per well into SDS-PAGE gels. Western blotting was performed as described in Supplemental Methods for CETSA experiments, except that blots were cut above the 50 kDa ladder band, the top half was probed with an anti-KCNQ1 C-terminal domain antibody (Alomone Labs, APC-022), and the bottom half was probed with an anti–β-actin antibody (Cell Signaling, 8457S) as a loading control. Bands corresponding to mycKCNQ1 (approximately 70 kDa) and β-actin were quantified with the Fiji/ImageJ BandPeakQuantification plug-in (77, 78), and mycKCNQ1 band intensities were normalized to the β-actin intensity in the same lane. Normalized mycKCNQ1 band intensities were then represented as a “percent remaining” from the 0-hour treatment time point and plotted using GraphPad Prism 10.

### Cellular thermal shift assay

The effects of compounds on the thermal stability of KCNQ1 in cells was assessed with a cellular thermal shift assay (CETSA). The principles and protocol for CETSA have been described in detail in several publications (79, 80), and CETSA has also been optimized for studies of membrane proteins more broadly (81), and for studying KCNQ1 in particular (12). Methods used in this work are described briefly in Supplemental Methods.

### Cell viability

Viability of cells after treatment with 0.1%–0.2% DMSO or 10–30 μM VU0494372 for 16 hours was assessed with trypan blue staining. Cells were detached with trypsin, retaining all media to ensure collection of all cells. Cell solutions were diluted 1:2 in trypan blue (Invitrogen, T10282), and then density and viability (percentage live cells) were determined with a Countess 3 FL (Invitrogen) automated cell counter. Cells were killed with approximately 50% ethanol and were assessed for viability as a control.

### Automated patch clamp recording

#### *Plasmids and site-directed mutagenesis*.

Full-length cDNA encoding WT KCNQ1 (GenBank accession number AF000571) were engineered in the mammalian expression vector pIRES2-EGFP (BD Biosciences Clontech) as previously described (74). This vector enabled expression of untagged channel subunits with fluorescent proteins as a means for tracking successful cell transfection.

#### *Electroporation*.

Plasmid encoding WT or mutant KCNQ1 was transiently transfected into Chinese hamster ovary (CHO) cells stably transfected with human KCNE1 (CHO-KCNQ1 cells) by electroporation using the MaxCyte STX system (MaxCyte Inc.) to generate WT I__Ks__-expressing cells, as previously described (74). Details are also included in Supplemental Methods.

#### *Electrophysiology*.

The day before automated patch clamp recording, electroporated cells were thawed. For analysis of chronic exposure of I__Ks__ cells to VU0494372, cells were grown for 10 hours, and then exposed to various concentrations of VU0494372 or vehicle (DMSO) for 16 hours. For analysis of acute exposure to VU0494372, cells were grown for 26 hours. Before experiments, cells were harvested using 0.25% trypsin. Aliquots were used to determine cell number and viability by automated cell counting and transfection efficiency by flow cytometry. Cells were then diluted to 300,000 cells/mL with external bath solution (see below) and allowed to recover 60 minutes at 15°C while shaking on a rotating platform. Chronic-exposure cells were continuously exposed to VU0494372 until the cell harvest step, during which compound was washed away and after which compound was no longer added. For acute exposure to VU0494372, untreated cells were exposed to various concentrations of VU0494372 or vehicle (DMSO) before whole-cell configuration was attained, and currents were recorded about 5 minutes after initiation of exposure. Automated patch clamp experiments were performed using the Syncropatch 384 platform (Nanion Technologies) and 4-hole, 384-well S-type (0.75–1.25 MΩ in control solutions) recording chips. Pulse generation and data collection were carried out with PatchController384 v1.3.0 and DataController384 v1.2.1 software (Nanion Technologies). Whole-cell currents were recorded at room temperature in the whole-cell configuration, filtered at 3 kHz, and acquired at 10 kHz. The access resistance and apparent membrane capacitance were estimated using built-in protocols. Because we used 4-hole recording chips, we could not perform series resistance (Rseries) compensation. The average Rseries for all 810 cells recorded for this study is 4.1 ± 0.3 MΩ (mean ± SEM). The average seal resistance (Rseal), capacitance, and Rseries for each experimental condition tested are listed in Supplemental Table 2.

The external bath solution contained 140 mM NaCl, 4 mM KCl, 2 mM CaCl__2__, 1 mM MgCl__2__, 10 mM HEPES, 5 mM glucose, at pH 7.4. The internal solution contained 60 mM KF, 50 mM KCl, 10 mM NaCl, 10 mM HEPES, 10 mM EGTA, 2 mM ATP-Mg, at pH 7.2. Whole-cell currents were elicited from a holding potential of –80 mV using 2,000-millisecond depolarizing pulses (from –80 mV to +60 mV in +10 mV steps, every 10 seconds) followed by a 2,000-millisecond step to –30 mV to analyze tail currents and channel deactivation rate. Nonspecific currents were eliminated by recording of whole-cell currents before and after addition of the I__Ks__ selective blocker JNJ-303 (2 μM; Tocris Bioscience). Only JNJ-303–sensitive currents and recordings with seal resistance ≥ 0.1 GΩ and Rseries < 10 MΩ were analyzed.

Data were analyzed and plotted as previously described (74). Details are included in Supplemental Methods. Whole-cell currents were normalized for membrane capacitance. Peak currents were recorded at 1,990 milliseconds after the start of the voltage pulse. The voltage-dependence of activation was calculated by fitting of the normalized conductance-voltage curves with a Boltzmann function (tail currents measured at –30 mV). Voltage values used for Boltzmann fits were corrected for the voltage drop at each testing potential. The average voltage drop at every testing potential for each condition tested is listed in Supplemental Tables 3 and 4.

### Computational binding site prediction for VU0494372

Computational docking was used to identify potential binding sites of VU0494372 to KCNQ1. Extended methods are described in Supplemental Methods. An initial conformer of VU0494372 was obtained in Structure Data File (SDF) format from the Vanderbilt Discovery Collection compound library managed by the Vanderbilt High Throughput Screening Core. VU0494372 was first blind-docked to 2 different KCNQ1 conformations from published structural models: PDB 8SIK (52) and PDB 7XNK (53). Only the KCNQ1 tetramer was used (without calmodulin or any ions), including transmembrane and cytosolic domains. We separately blind-docked VU0494372 to each KCNQ1 construct using DiffDock (54). Seven of the 10 poses predicted by DiffDock using 8SIK placed VU0494372 at a site partially overlapping with the known docking site of ML277. All of the predicted poses generated by DiffDock using 7XNK place VU0494372 inside the pore domain, likely owing to the large pocket formed in the open state of the pore. Using the docked poses generated by DiffDock with PDB 8SIK as starting positions, we applied a thorough targeted docking procedure to refine and score the poses. We used the BioChemicalLibrary ConformerGenerator (82) function to perform 2,000 conformer generation iterations and select the top 100 conformers. We generated a Rosetta parameters file and associated PDB files of the compound conformers and used the RosettaLigand (55, 56) small molecule docking protocol to generate 200 bound structural models for each of the DiffDock starting positions. We used the Score12 function with the restore_pre_talaris_2013_behavior flag as recommended for small molecule scoring (83). RosettaLigand calculates several protein- and ligand-specific energy score terms and combines them to determine an overall docking score (55). A more negative ligand interface score indicates a more energetically favorable binding energy. The most energetically favorable docked poses predict interactions with residues adjacent to those in the experimentally determined ML277 binding pocket.

### Resynthesis of VU0494372 and synthesis of analogs

Synthesis and characterization of 1-(4-methylpiperazin-1-yl)-3-(4-(2,4,4-trimethylpentan-2-yl)phenoxy)propan-2-ol (VU0494372), 1-morpholino-3-(4-(2,4,4-trimethylpentan-2-yl)phenoxy)propan-2-ol (VU0489336), and 1-(piperidin-1-yl)-3-(4-(2,4,4-trimethylpentan-2-yl)phenoxy)propan-2-ol (VU0963991) are described in Supplemental Methods. Solvents were obtained from commercial sources. Commercial reagents were used as received. The compounds were purified by Teledyne ISCO normal-phase column chromatograph system. Where necessary, preparative reverse-phase HPLC was conducted on a Gilson HPLC system using a Phenomenex Luna column (100 Å, 50 × 21.20 mm, 5 μm C18) with UV/Vis detection. ^^1^^H NMR spectra were recorded on a Bruker 400 MHz spectrometer and are reported relative to deuterated solvent signals. Data for ^^1^^H NMR spectra are reported as follows: chemical shift (δ ppm), multiplicity (s = singlet, d = doublet, t = triplet, q = quartet, quint = quintet, m = multiplet, br = broad, app = apparent), coupling constants (Hz), and integration. ^^13^^C NMR spectra were recorded on Bruker 100, 125, or 150 MHz spectrometers and are reported relative to deuterated solvent signals. Liquid chromatography/mass spectrometry was conducted and recorded on an Agilent Technologies 6130 Quadrupole instrument. High-resolution mass spectrometry was conducted and recorded at the Notre Dame Mass Spectrometry and Proteomics Facility on a micrOTOF II instrument (Bruker).

### Statistics

All statistical tests performed on data included in this publication were performed using GraphPad Prism version 8 or version 10. Normally distributed data were analyzed with a 2-tailed unpaired *t* test when 2 values were compared, and a 1-way ANOVA with Dunnett’s multiple-comparison test for follow-up when 3 or more values were compared. When data were not normally distributed (in the case of data normalized to the control value, for example), a Wilcoxon’s matched-pairs signed rank test was performed when 2 values were compared, and a Kruskal-Wallis test with Dunn’s multiple-comparison test for follow-up was performed when 3 or more values were compared. A *P* value less than 0.05 was considered significant. All graphs show data mean, and error bars represent standard deviation.

### Study approval

Given the use of cells throughout, study approval was not required.

### Data availability

All data values for all graphs included in this paper are reported in the Supporting Data Values file. All data and materials are available upon request. The custom multiple sequence alignment tool Multiple Sequence Iterative Comparator (MuSIC) used for analysis of some DNA sequencing results is available at https://doi.org/10.18131/h6hc6-n0j20

## Author contributions

KRCM, ALG, and CRS conceptualized the study. KRCM, CGV, ACCG, IMR, KMS, JAB, TPH, KRB, GAS, AGW, KVL, ALG, and CRS designed experiments and methods. KRCM, CGV, ACCG, IMR, MCW, TPH, and RRD conducted experiments. KRCM, CGV, ACCG, IMR, KMS, MCW, JAB, TPH, ELD, GAS, AGW, KVL, ALG, and CRS analyzed and interpreted data. JM, ALG, and CRS supervised the work. The first full draft of the manuscript was written by KRCM and CRS. KRCM, CGV, ACCG, IMR, KMS, MCW, TPH, KRB, AGW, JM, KVL, ALG, and CRS reviewed and edited the manuscript.

## Funding support

This work is the result of NIH funding, in whole or in part, and is subject to the NIH Public Access Policy. Through acceptance of this federal funding, the NIH has been given a right to make the work publicly available in PubMed Central.

- National Institutes of Health (NIH) grants 4T32GM065086-14 and F31HL168964-01 (to KRCM).
- NIH grant R01HL122010 (to ALG and CRS).
- NIH grants S10OD034362 and T32DK007061 (to ACCG).
- Deutsche Forschungsgemeinschaft (German Research Foundation) SFB1423, project 421152132 (to JM).
- Humboldt Professorship of the Alexander von Humboldt Foundation (JM).
- Vanderbilt Institute of Chemical Biology (to IMR, JAB, TPH, ELD, GAS, AGW, HTS Core Facility, and Vanderbilt University Medical Center Flow Cytometry Shared Resource).
- Vanderbilt Ingram Cancer Center P30CA68485 (to HTS Core Facility).
- NIH grant NCI R50CA211206 (to JAB).
- Vanderbilt Digestive Disease Research Center DK058404 (Vanderbilt University Medical Center Flow Cytometry Shared Resource).

## Supplementary Material

Supplemental data

Unedited blot and gel images

Supporting data values

## Version 1 01/08/2026

In-Press Preview

## Version 2 03/09/2026

Electronic publication
