# Supplementary material for: High-throughput screening identifies a trafficking corrector for long QT syndrome–associated KCNQ1 variants
Source: JCI Insight. 2026 Jan 8;11(5):e201297. doi: 10.1172/jci.insight.201297 (PMC13041670; doi:10.1172/jci.insight.201297)

Full, unedited blots for Figure 4B. Also shown in supplemental figure 7. All lanes from top half of blots shown in figure panel.

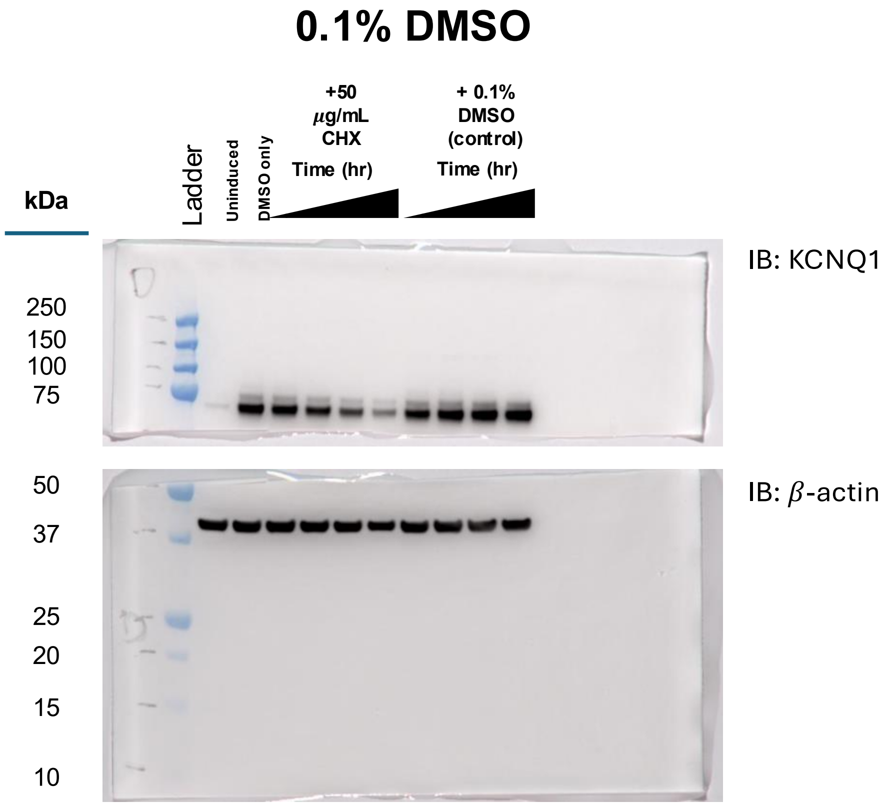

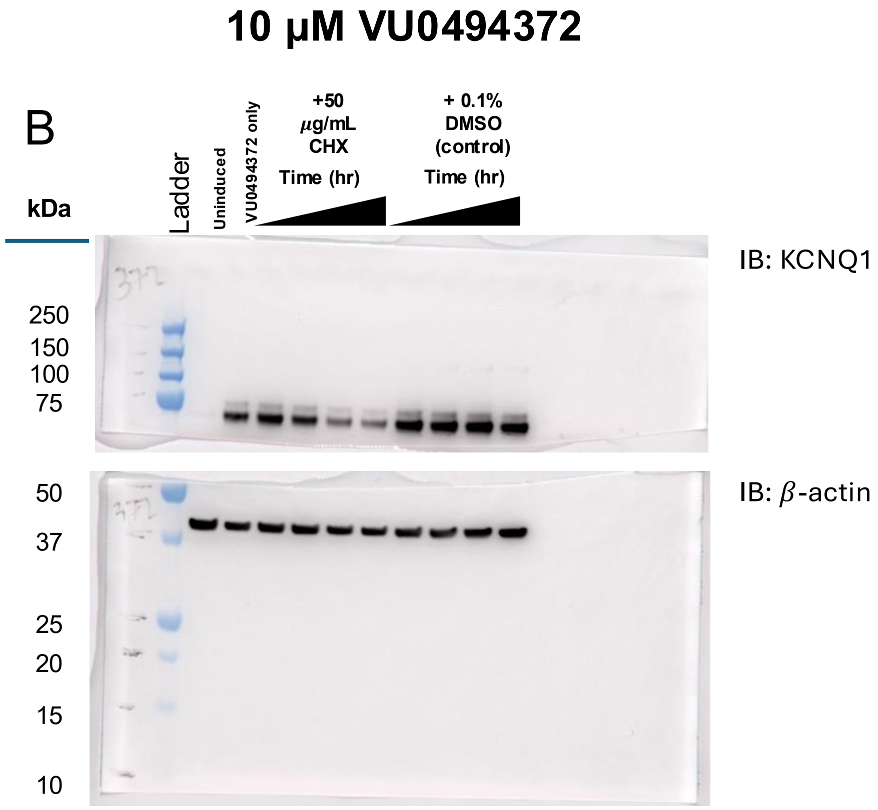

Full, unedited blot for Supplemental Figure 1B. Lanes highlighted in red box shown in figure panel.

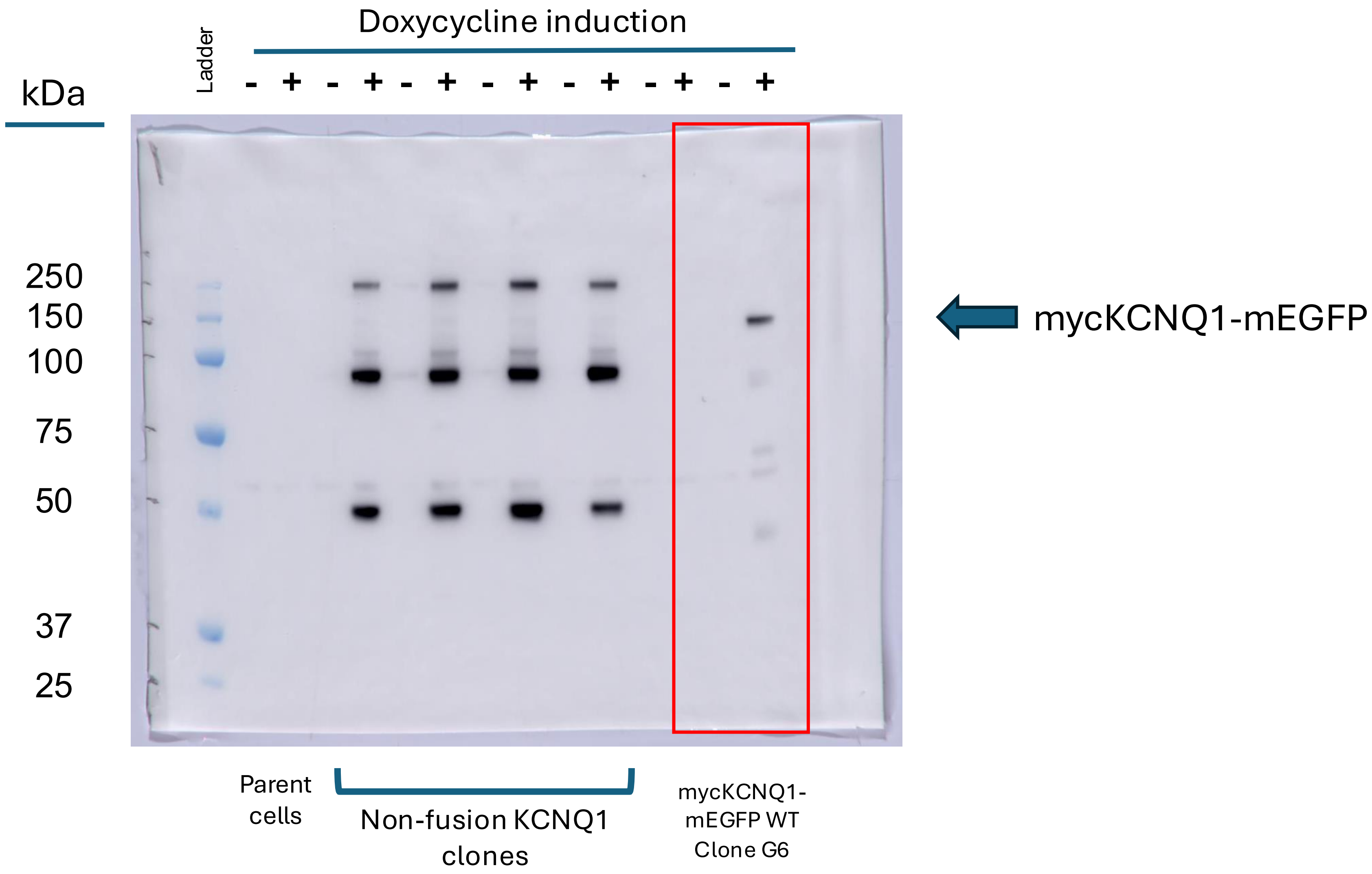

Supplement: Unedited blot and gel images [file jciinsight-11-201297-s129.pdf]
